# Social, demographic and health characteristics of men fathering children at different ages

**DOI:** 10.1038/s41598-021-00482-5

**Published:** 2021-10-25

**Authors:** Nina Kornerup, Anne-Marie Nybo Andersen, Per Kragh Andersen, Josephine Funck Bilsteen, Stine Kjaer Urhoj

**Affiliations:** 1 Faculty of Health and Medical Sciences, Section of Epidemiology, University of Copenhagen, Øster Farimagsgade 5A, Box 2099, 1014 Copenhagen K, Denmark; 2 Department of Gynecology and Obstetrics, Holbæk Hospital, Smedelundsgade 60, 4300 Holbæk, Denmark; 3 Faculty of Health and Medical Sciences, Section of Biostatistics, University of Copenhagen, Øster Farimagsgade 5 opg. B, P.O. Box 2099, 1014 Copenhagen K, Denmark

**Keywords:** Epidemiology, Risk factors, Epidemiology

## Abstract

The aim of this study was to examine associations between selected sociodemographic, socioeconomic, and health characteristics and the rates of fatherhood in different age groups. We investigated rates between 2011 and 2015 in a population-based register study including all men born from 1945 to 1995 residing in Denmark in 2011. The study population consisted of 1,867,108 men who fathered 268,612 children during the follow-up. The associations were quantified as incidence rate ratios using Poisson regression. Young men had higher rates of fathering a child if they lived outside the Capital Region, had a relatively high income, were previously diagnosed with cardiovascular disease, psychoactive substance abuse, personality disorders, schizophrenia or behavioural and emotional disorders. Men of advanced age had higher rates of fathering a child when born outside Denmark, living in the Capital Region, were in the lower or upper 10th percentile income group, were self-employed or unemployed or previously diagnosed with depression. Men of advanced age had lower rates of fathering a child if previously diagnosed with somatic diseases, psychoactive substance abuse or mental retardation. The findings highlight the importance of consideration of various sociodemographic, socioeconomic, and health characteristics when studying associations between paternal age and offspring health.

## Introduction

Maternal age and various socioeconomic and health characteristics are associated with adverse short- and long-term outcomes in their offspring^1,2^. Some studies have assessed the relationship between paternal age at conception and adverse outcomes in the offspring, but studies which investigated paternal socioeconomic and health characteristics and adverse short and long-term child outcomes are limited^3^.

Studies investigating maternal age at conception and adverse health outcomes in offspring find a U-shaped association between maternal age and various adverse outcomes in offspring, e.g. infants born to very young women and women at advanced age have greater risks for stillbirth, preterm birth, neonatal death, congenital anomalies, and low birth weight^2,4^.

Though fewer studies have been conducted, the same U-shaped association has been found when investigating paternal age and various adverse health outcomes in offspring^4–6^. This is particularly evident for mental illnesses in the child such as autism, autism spectrum disorders and schizophrenia, but is also found for stillbirth, congenital anomalies and orofacial clefts specifically^1,7–19^. To interpret and substantiate findings from such studies, it is of great importance to characterize men and women becoming parents at different ages as it has been speculated whether the U-shaped associations are partly due to (residual) confounding or selection forces whereby men with e.g. a certain genetic vulnerability reproduce earlier or later in life^13,20,21^.

Several studies characterizing women becoming mothers at different ages have been conducted^22–25^, but studies exploring such paternal age-related characteristics are lacking^26,27^. Thus, important knowledge concerning factors potentially confounding the associations or factors related to potential selection mechanisms might not be taken into consideration when examining findings of paternal age-related adverse health effects in the offspring.

With this study, we describe the distribution of various characteristics of fathers across all ages in order to contribute to the interpretation of results from studies investigating the association between paternal age and offspring health and to emphasize the importance of careful consideration of these characteristics in future studies. We selected various sociodemographic, socioeconomic, and health characteristics relevant to this discussion. The sociodemographic and socioeconomic characteristics were chosen to provide a general view of men who have children at different ages. The somatic and mental health characteristics were either selected based on studies showing a paternal age-related risk for these conditions in offspring, e.g. tumours, congenital anomalies and schizophrenia or to explore how rough proxies for lifestyle diseases are associated with the rate of fatherhood at different ages, e.g. endocrinological and cardiovascular diseases.

Many of the selected characteristics vary naturally between younger and older men. To avoid comparing these groups of men directly, we examined how the selected characteristics were associated with the rate of fatherhood within different age groups.

Thus, the aim of this study was to make a broad characterization of men fathering children at different ages by examining the association between selected sociodemographic, socioeconomic, and health characteristics and the rate of fatherhood in different age groups.

## Materials and methods

### Study population

This study was performed as a population-based register study including all men born from January 1, 1945 to December 31, 1995 and who were residing in Denmark on January 1, 2011. We investigated the rate of fathering a child in the period from January 1, 2011 to December 31, 2015, a total of five years. We included all men on January 1, 2011, regardless of parity, and followed them until December 31, 2015, regardless of whether they fathered a child or not (or whether they would father a child after December 31, 2015). In total, 1,867,108 men constituted the study population and 218,959 of these fathered 268,612 children during the five-year follow-up period.

The study population was divided into nine age groups according to their age on January 1, 2011: 15–19 years, 20–24 years, 25–29 years, 30–34 years, 35–39 years, 40–44 years, 45–49 years, 50–54 years, and 55–65 years.

### Data

Data were obtained from several Danish health and administrative registers. Since 1968, anyone living in Denmark is given a unique personal registration number, the so-called CPR (Central Personal Register) number^28^. This number is used to identify individuals in all official registers. Statistics Denmark extracted information from the various registers using the CPR number, pseudo-anonymised the CPR numbers to PNR (personal number) numbers and stored the data on a secure server for analysis. With the PNR, it was possible to link information about individuals from different registers.

We used data from registers held by the Danish Health Data Authority and Statistics Denmark, including the Civil Registration System, the Employment Classification Module, the Medical Birth Registry, the Danish National Patient Register (DNPR) and the Danish Psychiatric Central Research Register^28–34^. The processing and linkage of data were approved by the Danish Data Protection Agency (UCHP reference number: 514-0230/18-3000). Ethical approval or informed consent was not required for register-based studies according to Danish legislation.

Information about country of birth, region of residency, educational level, employment status, and income was obtained from the Civil Registration System, the Population Education Register, the Employment Classification Module, and the Income Statistics Register in Statistics Denmark^35–37^.

Place of birth was categorised as Denmark and other than Denmark. Region of residency (on January 1, 2011) was defined as one of the five administrative regions of Denmark: the Capital region, Other Zealand, Southern Denmark, Central Jutland, and Northern Jutland. The highest educational level attained (as of January 1, 2011) was categorised according to the Danish International Standard Classification of Education 15, D-ISCED-15, and was divided into primary and lower secondary school (ISCED 1 and 2, approximately 9–10 years of education), upper secondary school (ISCED 3, approximately 12–13 years of education), bachelor’s or equivalent (ISCED 5 and 6, approximately 15–16 years of education), and master’s and doctoral level (ISCED 7 and 8, often at least 17–18 years of education)^38^. Employment status was defined using the primary source of income in 2010 and categorised as employed, self-employed, student, retired (including disability pension), unemployed (persons registered as job-ready who received unemployment benefits or cash benefits more than half of the year in 2010), or unattached to the labour market (persons not registered as job-ready with no or a very low income from work, and no or very low income from unemployment benefits and cash benefits, and who are not in training/education). The individual’s disposable income in the period from January 1, 2010 to December 31, 2010 was divided into percentiles: lower 10th percentile, 10–25th percentile, 25–75th percentile, 75–90th percentile, and upper 90th percentile.

Information about medical history was obtained from the DNPR, which has registered all diagnoses given at hospitals since 1977. The International Classification of Diseases 8th Revision (ICD-8) and 10th Revision (ICD-10) were used for diagnoses. In Denmark, the ICD-8 was used from 1969 to 1993, and the ICD-10 from 1994 onwards. Men were classified as having a mental illness or a somatic disease if they had been admitted as an inpatient or had been in outpatient care before January 1, 2011. We included somatic and mental illnesses where a paternal age-related increased risk had previously been suggested^8,9^ or as rough proxies for lifestyle diseases. The following somatic diseases were included: malignant and benign tumours, cardiovascular diseases, endocrine diseases and congenital anomalies. The following mental illnesses were included: mental and behavioural disorders due to psychoactive substance uses (from here denoted psychoactive substance abuse), schizophrenia and related disorders, depression, bipolar disorders, personality disorders, mental retardation, pervasive developmental disorders, and behavioural and emotional disorders. The diseases can be seen in Supplementary Table I, together with the corresponding ICD-10 and ICD-8 codes for the included diagnoses.

### Statistical analyses

We investigated characteristics of men fathering a child at different ages. Older men will differ from younger men simply because they are older. As we were not interested in this difference, we investigated how selected sociodemographic, socioeconomic and health characteristics were associated with the rate of fatherhood in the predefined age groups, estimating incidence rate ratios (IRRs) using Poisson regression. Crude IRRs were estimated in each of the nine age groups according to the men’s age on January 1, 2011 and presented graphically. For all sociodemographic characteristics, the reference group is all men in that age group who are in a specific stratum of the variable, e.g., all men in regular employment. For mental illnesses and somatic diseases, the reference group is all men in that age group who had not received the specific diagnosis. The IRRs were not adjusted for other factors, as the analysis performed is a description of the relationship between the selected characteristics and fatherhood in different age groups—and not an attempt to establish any causal relationships. To evaluate if the IRRs for fatherhood relating to different characteristics differed between the various age groups, we performed a Wald test of interaction between age group and the specific characteristic.

The men in the study population entered the study on January 1, 2011 and were followed until death, emigration, or December 31, 2015, whichever came first. If a man fathered a child several times during the follow-up period, all births were counted in the rate.

All statistical analyses was carried out using STATA (StataCorp. 2017. Stata Statistical Software: Release 15. College Station, TX: StataCorp LLC).

## Results

Table 1 shows the distribution of selected sociodemographic, socioeconomic, and health characteristics within each age group of Danish men aged 15–65 years on January 1, 2011. More than 75% of the children were born to fathers 25–39 years of age.

**Table 1:** Sociodemographic and health characteristics according to the age of the man at January 1st 2011.

Age groups (years)	15–19	20–24	25–29	30–34	35–39	40–44	45–49	50–54	55–65
Number of births	6148	35,805	79,702	82,206	44,094	14,624	4409	1131	493
Total person years in the group	897,042.9	813,594.3	741,518.4	830,722.3	955,762.8	1,002,162	1,018,531	903,882	1,896,910
Total number of men	182,811	172,011	156,519	171,725	195,450	204,182	207,489	184,743	392,178
Place of birth (%)									
Denmark	89.4	83.8	81.0	85.0	88.3	88.6	89.7	91	93.9
Other than Denmark	10.6	16.2	19.0	15.0	11.7	11.4	10.4	9	6.1
Region of residents (%)									
Capital Region	26.2	31.3	35.3	34.3	32.2	30.0	29.1	27.6	26.1
Other Zealand	15.6	11.7	10.5	12.2	14.4	15.6	15.6	15.8	16.7
Southern Denmark	23.4	20.5	19.6	20.2	20.7	21.8	22.2	22.8	22.9
Central Jutland	23.8	25.5	24.2	23.3	23.0	22.6	22.4	22.8	23.0
Northern Jutland	11.1	11.0	10.4	10.0	9.8	10.1	10.7	11	11.3
Educational level (%)									
Primary and lower secondary	99.9	50.2	24.6	19.5	19.5	21.7	23.3	25.5	25.0
Upper secondary	0.1	47.8	52.6	47.4	47.0	48.5	50.1	48.3	49.2
Bachelor’s degree	0.0	2.0	17.5	20.2	20.8	19.6	17.7	17.4	17.4
Master’s degree/doctoral level	0	0.1	5.3	12.8	12.7	10.3	8.9	8.9	8.4
Employment (%)									
Employed	9.2	46.6	63.3	75.5	77.5	76.1	74.4	72.7	55.6
Self-employed	0.1	1.2	3.2	5.1	6.6	7.6	8.1	8.2	8.0
Student	83.5	34.4	15.7	3.3	1.0	0.4	0.2	0.1	0.0
Unemployed	1.1	5.6	9.1	9.2	8.2	8.1	7.9	7.5	5.0
Retired	0.3	1.4	2.1	2.7	3.6	5.1	6.9	9.3	28.8
Unattached to the labour market	5.8	10.9	6.7	4.3	3.1	2.8	2.5	2.3	2.6
Disposable income (percentiles)^a^									
Lower 10th	− 153^b^	11,309	9849	8755	16,852	2858	− 8831^b^	10,028	26,482
10–25th	2355	50,888	79,869	123,832	145,075	149,903	151,239	152,853	143,793
25–75th	21,925	99,982	162,703	212,242	235,732	243,569	245,090	244,465	226,124
75–90th	58,148	167,453	237,903	292,145	333,890	356,689	363,021	362,075	343,766
Upper 90th	111,279	233,455	322,297	434,158	574,505	675,938	734,322	719,476	689,028
Somatic diseases (%)^c^									
Malignant and benign tumours	3.8	3.9	4.5	5.4	6.4	7.8	9.4	11.4	16.9
Cardiovascular diseases	2.0	3.1	4.1	6.0	8.2	11.6	15.8	21.6	33.2
Endocrine diseases	4.7	4.7	4.6	4.0	4.2	5.3	7.1	9.9	15.2
Congenital anomalies	12.2	11.6	11.2	8.7	7.0	5.3	3.9	3.1	2.7
Mental illnesses (%)^d^									
Psychoactive substance abuse	2.1	5.4	6.9	6.9	6.1	6.2	6.6	7.1	6.7
Schizophrenia and related disorders	0.6	1.3	1.7	1.8	1.8	1.7	1.7	1.6	1.2
Depression	1.0	1.7	2.1	2.4	2.5	2.7	2.8	2.9	2.8
Bipolar disorders	0.0	0.1	0.2	0.3	0.3	0.3	0.4	0.5	0.5
Personality disorders	0.2	0.9	1.5	1.8	1.7	1.5	1.5	1.2	0.8
Mental retardation	1.2	1.1	0.8	0.6	0.5	0.4	0.4	0.4	0.3
Pervasive developmental disorders	2.1	1.1	0.5	0.2	0.1	0.1	0.1	0.1	0.0
Behavioural and emotional disorders	6.8	5.2	3.6	2.5	2.0	1.8	1.7	1.5	1.1

Results are according to conditions that prevailed in 2010 for all men aged 15–65 years living in Denmark January 1, 2011 divided into age groups.

Numbers are shown as percentage distribution unless described otherwise.

^a^Disposable income is shown as the average disposable income (rounded) for the year 2010 for each age group divided into percentiles: lower 10th percentile, 10–25th percentile, 25–75th percentile, 75–90th percentile, and upper 90th percentile. Disposable income is income after taxes, interests, and rental value of own home.

^b^Disposable income can be negative, e.g. due to negative interests.

^c^Percentage distribution of somatic diseases diagnosed before January 1, 2011.

^d^Percentage distribution of mental illnesses diagnosed before January 1, 2011.

**Figure 1:**
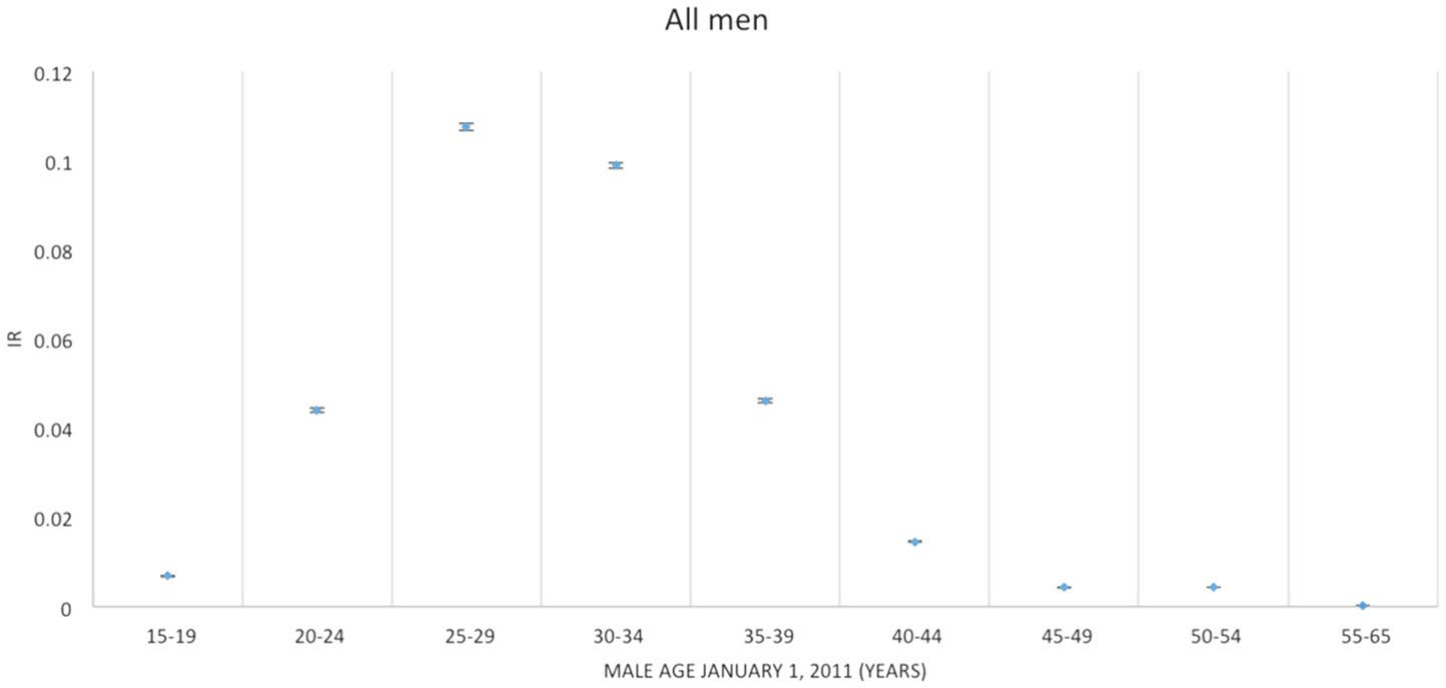
Incidence rates (IR) of fathering a child according to age^1^ in each age group for the entire study period. Men aged 15–65 years living in Denmark January 1, 2011 to December 31, 2015. ^1^Age according to the man’s age by January 1, 2011 divided into age groups.

Figure 1 shows the incidence rates (IRs) of fathering a child in each age group for the entire study period. The highest rates of fathering a child were seen in the age groups 25–29 years and 30–34 years and the lowest rates of fathering a child were seen in the age groups 15–19 years and 40–65 years.

**Figure 2:**
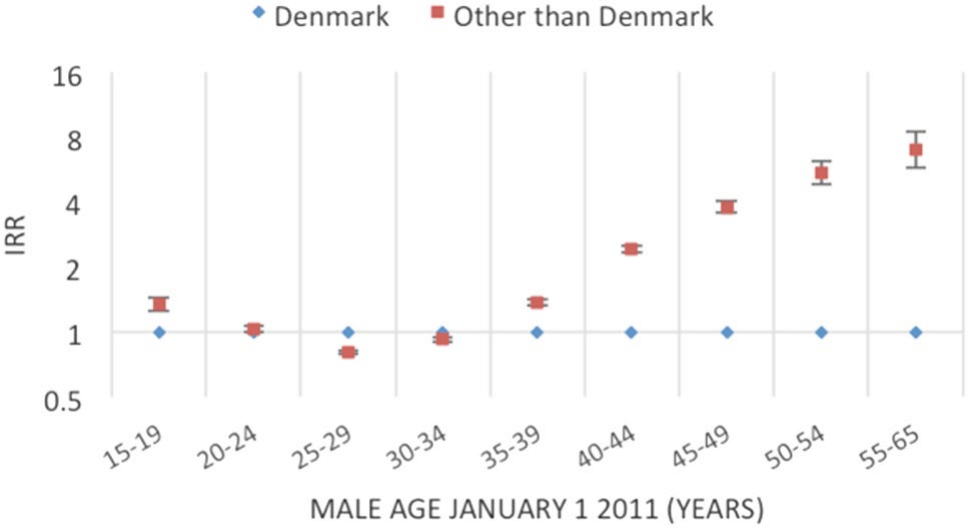
Incidence rate ratios (IRR) of fathering a child according to place of birth^1^ and age^2^. Men aged 15–65 years living in Denmark January 1, 2011 to December 31, 2015. ^1^Place of birth was categorised as: Denmark or other than Denmark. ^2^Age groups according to the man’s age by January 1, 2011.

**Figure 3:**
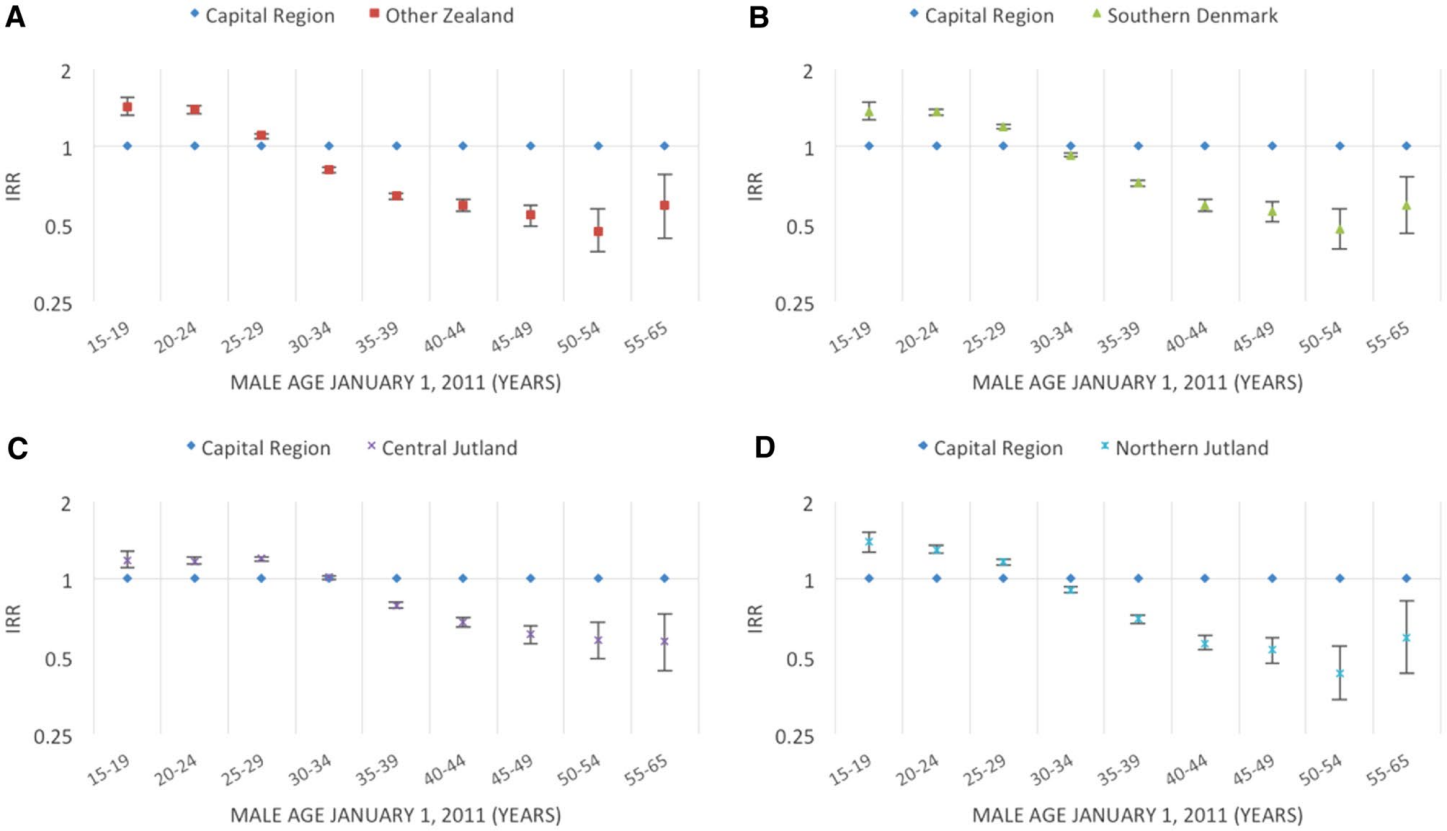
Incidence rate ratios (IRR) of fathering a child according to region of residency^1^ and age^2^. Men aged 15–65 years living in Denmark January 1, 2011 to December 31, 2015. ^1^Region of residency by January 1, 2011 categorised into one of the five administrative regions of Denmark: the Capital Region, Other Zealand, Southern Denmark, Central Jutland or Northern Jutland. ^2^Age groups according to the man’s age by January 1, 2011.

**Figure 4:**
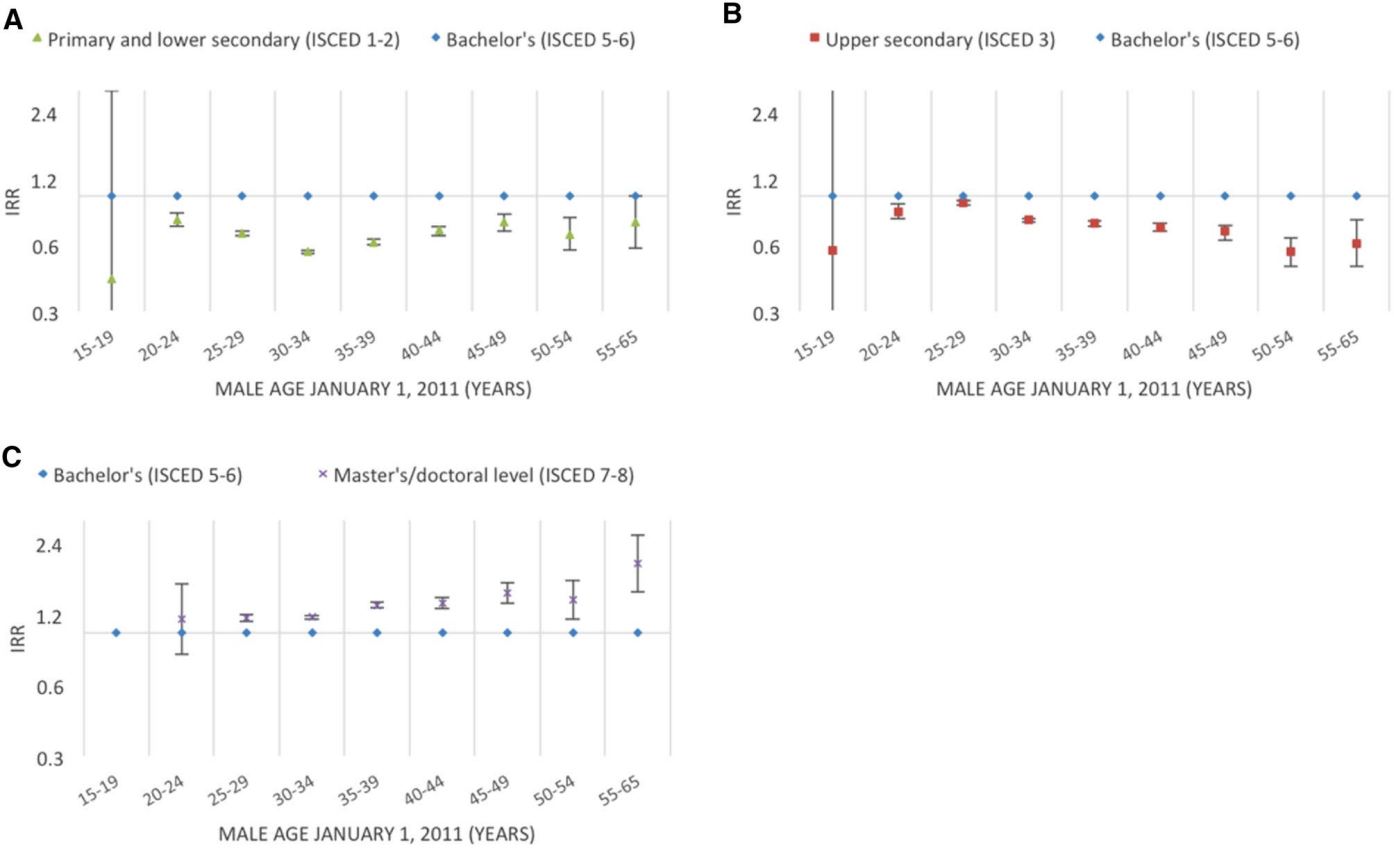
Incidence rate ratios (IRR) of fathering a child according to educational level^1^ and age^2^. Men aged 15–65 years living in Denmark January 1, 2011 to December 31, 2015. ^1^Highest educational level attained (as of January 1, 2011) was categorised according to the Danish International Standard Classification of Education 15, D-ISCED-15, and divided into primary and lower secondary (ISCED 1–2), upper secondary (ISCED 3), bachelor’s degree or equivalent (ISCED 5–6), and master’s/doctoral level (ISCED 7–8). No men in the group 15–19 years had a master’s/doctoral level education, thus the IRRs in this age group is not estimated. ^2^Age groups according to the man’s age by January 1, 2011.

**Figure 5:**
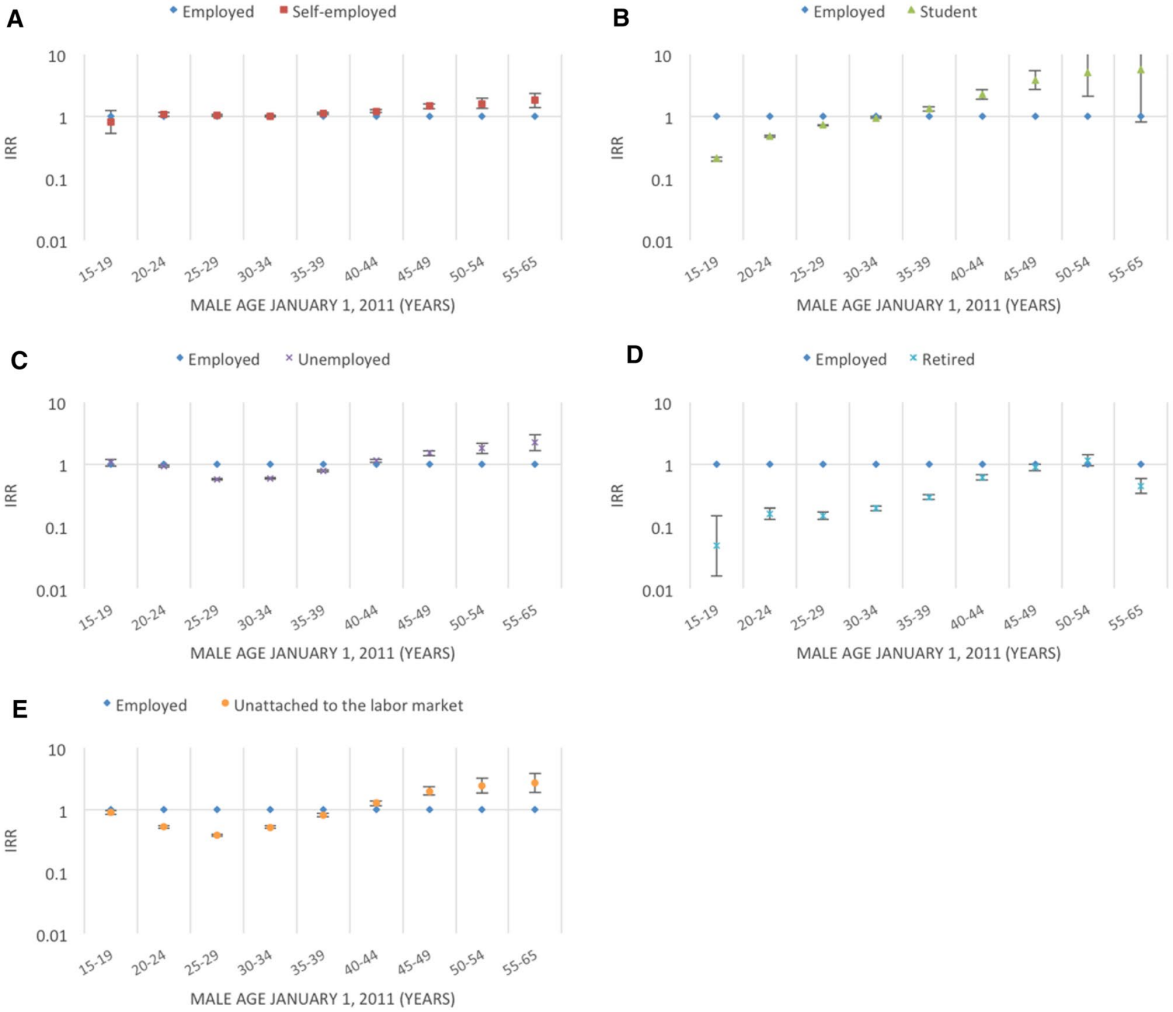
Incidence rate ratios (IRR) of fathering a child according to employment status^1^ and age^2^. Men aged 15–65 years living in Denmark January 1, 2011 to December 31, 2015. ^1^Employment status defined from the primary source of income in 2010 and categorised as: employed, self-employed, student, unemployed, retired, or unattached to the labour market. 2Age groups according to the man’s age by January 1, 2011.

**Figure 6:**
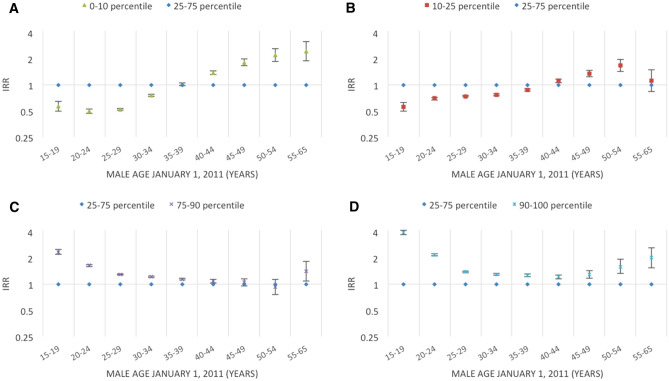
Incidence rate ratios (IRR) of fathering a child according to average income^1^ and age^2^. Men aged 15–65 years living in Denmark January 1, 2011 to December 31, 2015. ^1^The individual’s disposable income in the period from January 1, 2010 to December 31, 2010 divided into percentiles: lower 10th percentile, 10–25th percentile, 25–75th percentile, 75–90th percentile, and upper 90th percentile. ^2^Age groups according to the man’s age by January 1, 2011.

**Figure 7:**
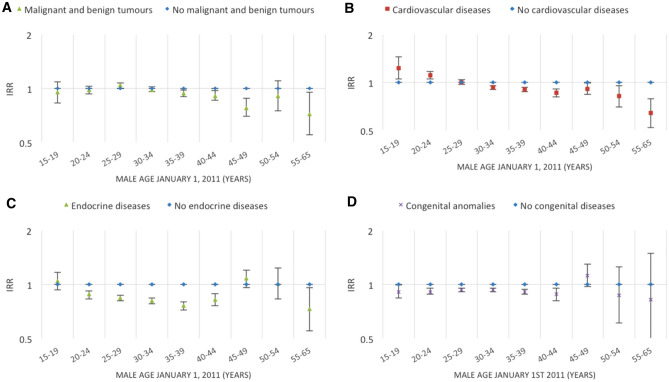
Incidence rate ratios (IRR) of fathering a child according to selected somatic diseases^1^ and age^2^. Men aged 15–65 years living in Denmark January 1, 2011 to December 31, 2015. ^1^Somatic diseases (diagnosed before January 1, 2011) included malignant and benign tumours, cardiovascular diseases, endocrine diseases, or congenital anomalies. ^2^Age groups according to the man’s age by January 1, 2011.

**Figure 8:**
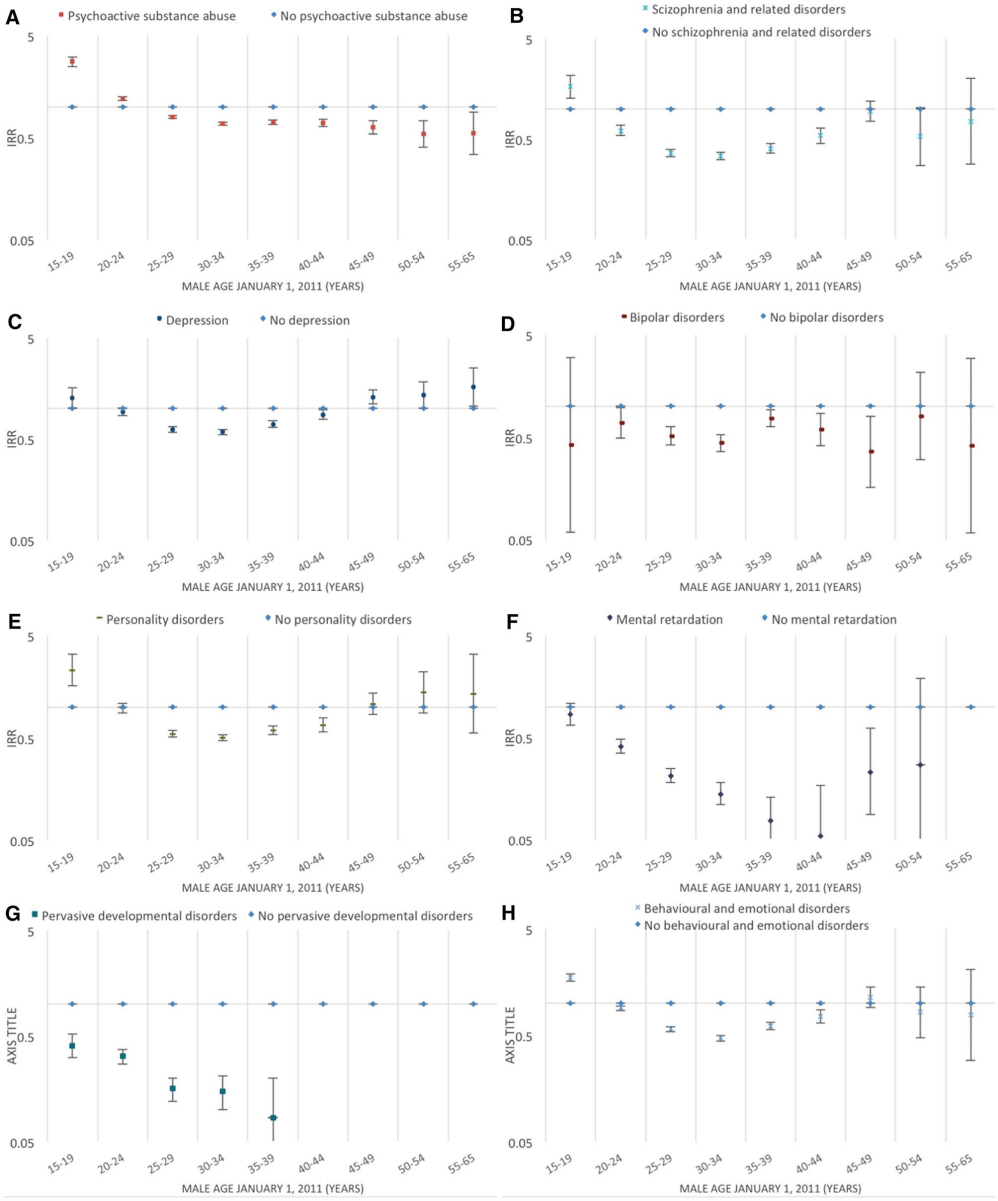
Incidence rate ratios (IRR) of fathering a child according to selected mental illnesses^1^ and age^2^. Men aged 15–65 years living in Denmark January 1, 2011 to December 31, 2015. ^1^Mental illnesses (diagnosed before January 1, 2011) included: psychoactive substance abuse, schizophrenia and related disorders, depression, bipolar disorders, personality disorders, mental retardation, pervasive developmental disorders, and behavioural and emotional disorders. No men became fathers in the age groups 40–65 years for pervasive developmental disorders or in the age group 55–65 years for mental retardation, thus the IRRs in these age groups are not estimated. ^2^Age groups according to the man’s age by January 1, 2011.

Figures 2, 3, 4, 5, 6, 7 and 8 show the IRRs of fathering a child in each age group according to a specific sociodemographic characteristic, somatic disease or mental illness compared with the reference group. The IRRs and the corresponding confidence intervals can be seen in the Supplementary Table II.

### Socioeconomic characteristics

Men born in countries outside Denmark had the highest rates of fathering a child in the youngest- and oldest age groups compared with men born in Denmark (Fig. 2); from age 35 years, the IRRs were higher with higher age, reaching a sevenfold higher rate (IRR = 6.97, 95% CI 5.75–8.43) of fathering a child in the oldest age group.

Compared with men living in the Capital Region, men in all other regions had higher rates of fathering a child at young ages (15–29 years) and lower rates for fathering a child at older ages (35 + years) (Fig. 3A–D).

In general, compared with men with a bachelor’s degree or equivalent, men in all age groups had a lower rate of fathering a child if their educational level was primary/lower secondary school or upper secondary school (Fig. 4A,B). Men with a master’s or doctoral level education had a higher rate of fathering a child from age 25 years and older; at age 55–65 years, the rate was almost doubled (IRR = 1.97, 95% CI 1.49–2.61) (Fig. 4C).

Men at an advanced age (≥ 35 years) that were self-employed had higher rates of fathering a child compared with men in regular employment; the highest IRR was seen for men aged 55–65 years (IRR = 1.81, 95% CI 1.39–2.34) (Fig. 5A). For men who were unemployed or unattached to the labour market, lower rates of fathering a child at a younger age (≤ 39 years) and higher rates of fathering a child at an older age (≥ 40 years) were seen (Fig. 5C,E). Retired men had, in general, lower rates of fathering a child (Fig. 5D). Students had lower rates of fathering a child at a young age (≤ 34 years) and higher rates of fathering a child at an advanced age (≥ 35 years) (Fig. 5B). Wide confidence intervals were seen for students of advanced age, because there are few students and very few men fathered a child in these age groups.

Compared to men with a yearly disposable income corresponding to the 25–75th percentile, men with a lower income had lower rates of fathering a child at a young age (15–34 years) and higher rates of fathering a child at older ages (40 + years) (Fig. 6A,B). In general, men with a higher income had higher rates of fathering a child at all ages (Fig. 6C,D).

### Health characteristics

#### Somatic diseases

For men previously diagnosed with a somatic disease, the rate of fathering a child was, in general, lower than or similar to the rate for men who had not previously been diagnosed with the diagnosis in question (Fig. 7A–D). This was, however, not the case for young men (15–24 years) who were previously diagnosed with a cardiovascular disease; these men had a slightly higher rate of fathering a child compared with men who had not received a diagnosis for a cardiovascular disease (Fig. 7B).

#### Mental illnesses

Men previously diagnosed with a psychoactive substance abuse had higher rates of fathering a child at young ages compared with men who were not previously diagnosed with a psychoactive substance abuse (15–19 years IRR = 2.77, 95% CI 2.48–3.09; 20–24 years IRR = 1.20, 95% CI 1.18–1.29). Among men above 24 years, rates of fathering a child were lower in all age groups (Fig. 8A).

Concerning schizophrenia and other related disorders, men who had received such diagnoses had a higher rate of fathering a child at age 15–19 years (IRR = 1.66, 95% CI 1.27–2.15) and a similar rate at age 45–49 years compared with men without such diagnoses. In all other age groups, lower rates of fathering a child were seen. Among the oldest fathers (≥ 50 years), the IRRs were estimated with a large uncertainty, as few men diagnosed with schizophrenia and related disorders fathered a child in these age groups (Fig. 8B).

Men previously diagnosed with depression had lower rates of fathering a child at the age 20–44 years compared with men who had not earlier been diagnosed with depression. For men ≤ 19 years and ≥ 45 years, the rates were higher. The incidence rate was 62% higher (IRR = 1.62, 95% CI 1.05–2.51) for men aged 55–65 years (Fig. 8C).

Higher rates of fathering a child at age 15–19 years were seen for men previously diagnosed with personality disorders (Fig. 8E) or behavioural and emotional disorders (Fig. 8H) compared with men who had not previously received the diagnoses in question (IRR = 2.31, 95% CI 1.62–3.29 and IRR = 1.73, 95% CI 1.60–1.88). For men aged 25–44 years, the rates were lower, and, for men aged ≥ 45 years, the rates were similar to the rates seen in men who were not previously diagnosed with a personality disorder or behavioural and emotional disorders.

Men previously diagnosed with bipolar disorder (Fig. 8D), mental retardation (Fig. 8F), or pervasive developmental disorders (Fig. 8G) had lower rates of fathering a child in all age groups compared with men who had not earlier received the diagnosis in question (Fig. 8D,F,G). Very few men with these diagnoses fathered a child when above the age of 40 years (for pervasive developmental disorders none in the age group 40–65 years and for mental retardation none in the age group 55–65 years) and, therefore, the IRRs in these age groups were either not estimated or estimated with great uncertainty.

## Discussion

This study showed that the rate of fathering a child was highest for men aged 25–34 years and that the rate at different ages varied according to sociodemographic- and socioeconomic factors and health characteristics among men living in Denmark in 2011–2015.

Men in this age group, 25–34 years, had higher rates of fathering a child if they had a bachelor or higher educational level and if they were working or studying and had an income within the 25–75th percentile or higher. Concerning mental health, for all diagnosis included, the rates of fathering a child in this age group were lower if previously diagnosed with a mental illness, than if not previously diagnosed with a mental illness.

Since our study is a descriptive study, the results cannot explain mechanisms behind the differences in the rates of fathering a child. It is, however, worth highlighting that men diagnosed with a mental illness in general have a lower rate of fathering a child in the ages where most men become fathers. We can only speculate on the reasons for this, e.g. whether it is an active choice or involuntary, but knowledge about these mechanisms may form the basis for initiatives directed to improve health and quality of life for men suffering from mental illness and might alter the rates of fatherhood for this group of men.

We found that young men (≤ 24 years) who lived outside the capital region and young men who had a relatively high income had higher rates of fathering a child than those living in the capital region and those who had a lower income, respectively. In the youngest age group (≤ 19 years) those born outside of Denmark had higher rates of fathering a child than those born in Denmark. Moreover, we found that young men previously diagnosed with cardiovascular disease had a higher rate of fathering a child, which was not seen among men aged 25–34 years. This group of young men likely consisted mainly of men with congenital heart disease, who may have had to deal with difficulties in life and many hospitalizations due to their disease. This may contribute to an earlier maturation, but other mechanisms behind may be present. No studies investigating family planning in young men with congenital heart disease were found to confirm this hypothesis.

We also found that men were more likely to father a child in the very young age group (≤ 19 years) if they had previously been diagnosed with psychoactive substance abuse, personality disorders, schizophrenia, or behavioural and emotional disorders. A previous study investigating mental health in offspring find that offspring of young mothers, and to a lesser degree also young fathers, have a higher risk of developing mental illness, e.g. psychoactive substance abuse, hyperkinetic disorders, mental retardation, lower mean intelligence scores, and psychological disorders^8^. It is well known that mental illness in parents has negative health impact in children^39–42^. Various factors may contribute to this association, including factors related to age. Studies investigating the circumstances and mechanisms underlying this association could potentially lead to a better prevention of adverse outcomes in the offspring.

A higher rate of fathering a child at an advanced age (≥ 35 years) was seen among men who lived in the Capital Region, men who had the lowest and highest income, and men who were either self-employed or unemployed. In correspondence with the results of our study, a Norwegian study from 2013 showed that men who became fathers for the first time at an advanced age formed a heterogeneous group, in which the majority had a stable income and social situation and a minority were characterised by a low educational level, unemployment or single status^26^. This suggests that there might be two groups of men, in terms of sociodemographic and socioeconomic factors, who father a child at an advanced age; men with a high educational level and a relatively high income, and men with a low educational level, with no or little attachment to the labour market and a relatively low income.

In our study, we did not only include first time fathers because negative health effects in offspring have been found in studies including children fathered by men of advanced age regardless of whether the child was the first, second or third or more child. However, we were not able to include details about the number of previous children. Future studies examining the above mentioned two groups of men in relation to the number of previous children and how adverse outcomes in offspring are distributed between these two groups could contribute to the discussion about underlying mechanisms, including potential selection forces related to late fatherhood.

In terms of health characteristics, a higher rate of fathering a child at an advanced age was also seen among men who were previously diagnosed with depression. This trend may reflect the postponement of parenthood in men previously diagnosed with a depression; however, as we investigated the rate of men fathering a child regardless of whether the child was the first, second or later child, this study cannot shed light on this aspect. Postponement of parenthood is a rising tendency and further studies investigating differences in the rate of fathering a child at an advanced age and the underlying mechanisms could contribute with important knowledge.

Several of the characteristics were associated with a lower rate of fatherhood in the older age group, e.g. the somatic diseases and psychoactive substance abuse. A previous diagnosis of schizophrenia or related disorders likewise seemed to be associated with a lower rate of fatherhood at an advanced age, which is interesting as studies show an association between advanced paternal age and schizophrenia in children^9,43^.

Strengths of this study are the nationwide coverage, including all men aged 15–65 years living in Denmark on January 1, 2011, and the large sample size. Another methodological strength is the design of the analysis, enabling us to compare the rate of fatherhood according to specific characteristics across age groups. Because preconditions for many sociodemographic, socioeconomic and health characteristics are closely linked to age, e.g. income and cardiovascular diseases, it is not possible to directly compare younger and older men. To overcome this, we investigated the rate of fathering a child in different age groups according to the included sociodemographic, socioeconomic and health characteristics and thus estimated incidence rate ratios that were comparable across age groups.

The weaknesses of our study include the restriction to variables that Statistics Denmark or other administrative registers routinely collect, including DNPR. DNPR was established in 1976 and we had access to data on diagnoses from 1977 onwards. As a consequence, men receiving a diagnosis before 1977 appeared as if they had never received the concerned diagnosis—unless they had been registered with this diagnosis in relation to later contact with the healthcare system. This misclassification is only possible for men born before 1977 and we expect it to be largest for men with non-chronic diseases, including depression, psychoactive substance abuse and a variety of somatic diseases. It is, however, unlikely that such diseases not registered in the healthcare system for more than 30 years would have a major impact on the results, though this cannot be ruled out. There is also a more general risk of misclassification of the mental illnesses and somatic diseases. Men are in general consulting a primary care physician less frequently than women and are also less likely to seek help for mental health problems^44,45^. As a consequence, a general underdiagnoses might be present, which would dilute the differences between men with and without the diagnosis.

The sociodemographic- and socioeconomic characteristics were defined as per 2010, including region of residents, educational level, employment status and disposable income. These characteristics may have changed shortly before 2010 or during the five-year study period, which may also result in misclassification of the characteristics and should be taken into account when interpreting the results.

Men are less likely to seek help for mental health problems even when they are experiencing significant levels of psychological distress; Another limitation related to the diagnosing of the included diseases arises when we compare the IRRs across age groups. We performed a Wald test of interaction between age group and the specific characteristic, but, due to the difference in the number of years where a diagnosis could be registered and due to the fact that the diagnosing of some of the included diseases, e.g. behavioural and emotional disorders, has changed during the period from 1977 to 2010, it is difficult to conclude whether the significant interaction analysis estimates are due to circumstances related to the diagnosing or due to a real difference in the relationship between a diagnosis and fathering a child across age groups.

## Conclusion

The results show that the rate of fathering a child in different age groups varied according to sociodemographic, socioeconomic, and health characteristics of men living in Denmark in 2011.

It is beyond the scope of this study to draw conclusions about risk factors for offspring of fathers at different ages or recommendations for men becoming fathers, even though these are both needed and important. Our findings do, however, point to characteristics that are important in relation to the age-related rate of fatherhood and the interpretation of results from studies investigating the association between paternal age and offspring health. Moreover, the results highlight the importance of careful consideration of various sociodemographic, socioeconomic, and health characteristics in future studies. Factors associated with parenthood that may have negative health consequences for offspring are important for the general population and for couples considering parenthood.

## Supplementary Information

Supplementary Information.

## References

[1] Lean SC, Derricott H, Jones RL, Heazell AEP (2017). Advanced maternal age and adverse pregnancy outcomes: A systematic review and meta-analysis. PLoS ONE.

[2] Amjad S (2019). Social determinants of health and adverse maternal and birth outcomes in adolescent pregnancies: A systematic review and meta-analysis. Paediatr. Perinat. Epidemiol..

[3] Oldereid N (2018). The effect of paternal factors on perinatal and paediatric outcomes: A systematic review and meta-analysis. Hum. Reprod. Update.

[4] Thompson JA (2020). The risks of advancing parental age on neonatal morbidity and mortality are U- or J-shaped for both maternal and paternal ages. BMC Pediatr..

[5] Fang Y (2020). Effect of paternal age on offspring birth defects: A systematic review and meta-analysis. Aging.

[6] Khandwala YS (2018). Association of paternal age with perinatal outcomes between 2007 and 2016 in the United States: Population based cohort study. BMJ.

[7] D’onofrio BM (2014). Paternal age at childbearing and offspring psychiatric and academic morbidity. JAMA Psychiat..

[8] Jin J (2014). A comprehensive assessment of parental age and psychiatric disorders. JAMA.

[9] Malaspina D, Gilman C, Kranz TM (2015). Paternal age and mental health of offspring. Fertil. Steril..

[10] Wu S (2017). Advanced parental age and autism risk in children: A systematic review and meta-analysis. Acta Psychiatr. Scand..

[11] De Kluiver H, Buizer-Voskamp JE, Dolan CV, Boomsma DI (2017). Paternal age and psychiatric disorders: A review. Am. J. Med. Genet. Part B Neuropsychiat. Genet..

[12] Malaspina D (2001). Advancing paternal age and the risk of schizophrenia. Arch. Gen. Psychiatry.

[13] Weiser M (2020). Understanding the association between advanced paternal age and schizophrenia and bipolar disorder. Psychol. Med..

[14] Nybo Andersen A-M, Urhoj SK (2017). Is advanced paternal age a health risk for the offspring?. Fertil. Steril..

[15] Urhoj S, Andersen P, Mortensen L, Davey Smith G, Nybo Andersen A-M (2017). Advanced paternal age and stillbirth rate: A nationwide register-based cohort study of 944,031 pregnancies in Denmark. Eur. J. Epidemiol..

[16] Urhoj SK, Mortensen LH, Nybo Andersen AM (2015). Advanced paternal age and risk of musculoskeletal congenital anomalies in offspring. Birth Defects Res. Part B Dev. Reprod. Toxicol..

[17] Yang Q (2007). Paternal age and birth defects: How strong is the association?. Hum. Reprod..

[18] Berg E, Lie RT, Sivertsen Å, Haaland ØA (2015). Parental age and the risk of isolated cleft lip: A registry-based study. Ann. Epidemiol..

[19] Green RFMMSP (2010). Association of paternal age and risk for major congenital anomalies from the national birth defects prevention study, 1997 to 2004. Ann. Epidemiol..

[20] Bray I, Gunnell D, Davey Smith G (2006). Advanced paternal age: How old is too old?. J. Epidemiol. Commun. Health.

[21] Crow JF (2003). Development: There's something curious about paternal-age effects. Science.

[22] Fitzpatrick KE, Tuffnell D, Kurinczuk JJ, Knight M (2017). Pregnancy at very advanced maternal age: A UK population-based cohort study. BJOG Int. J. Obstet. Gynaecol..

[23] Nilsen ABV, WaldenstrÖM U, Hjelmsted A, Rasmussen S, Schytt E (2012). Characteristics of women who are pregnant with their first baby at an advanced age. Acta Obstet. Gynecol. Scand..

[24] Molina Cartes, R. & González Araya, E. Vol. 22 *Endocrine Development* 302–331 (S. Karger AG, Basel, Switzerland, 2012). 10.1159/000326706 22846537

[25] Guedes M, Canavarro MC (2014). Characteristics of primiparous women of advanced age and their partners: A homogenous or heterogenous group?. Birth.

[26] Nilsen ABV, Waldenström U, Rasmussen S, Hjelmstedt A, Schytt E (2013). Characteristics of first-time fathers of advanced age: A Norwegian population-based study. BMC Pregnancy Childbirth.

[27] Paavilainen M, Bloigu A, Hemminki E, Gissler M, Klemetti R (2016). Aging fatherhood in Finland: First-time fathers in Finland from 1987 to 2009. Scand. J. Public Health.

[28] Pedersen CB (2011). The danish civil registration system. Scand. J. Public Health.

[29] Thygesen LC, Daasnes C, Thaulow I, Brønnum-Hansen H (2011). Introduction to Danish (nationwide) registers on health and social issues: Structure, access, legislation, and archiving. Scand. J. Public Health.

[30] Sortsø C, Thygesen LC, Brønnum-Hansen H (2011). Database on Danish population-based registers for public health and welfare research. Scand. J. Public Health.

[31] Knudsen LB, Olsen J (1998). The danish medical birth registry. Dan. Med. Bull..

[32] Lynge E, Sandegaard JL, Rebolj M (2011). The danish national patient register. Scand. J. Public Health.

[33] Mors O, Perto GP, Mortensen PB (2011). The danish psychiatric central research register. Scand. J. Public Health.

[34] Bliddal M, Broe A, Pottegård A, Olsen J, Langhoff-Roos J (2018). The danish medical birth register. Off. J. Eur. Epidemiol. Federation.

[35] Jensen VM, Rasmussen AW (2011). Danish education registers. Scand. J. Public Health.

[36] Petersson F, Baadsgaard M, Thygesen LC (2011). Danish registers on personal labour market affiliation. Danish Reg. Pers. Labour Mark. Aff..

[37] Baadsgaard M, Quitzau J (2011). Danish registers on personal income and transfer payments. Danish Reg. Pers. Income Transf. Payments.

[38] www.dst.dk. https://www.dst.dk/da/Statistik/dokumentation/nomenklaturer/disced-15--uddannelsesniveau--fuldfoerte-uddannelser, 2018).

[39] Stein AP (2014). Effects of perinatal mental disorders on the fetus and child. The Lancet (British edition).

[40] Rasic D, Hajek T, Alda M, Uher R (2013). Risk of mental illness in offspring of parents with schizophrenia, bipolar disorder and major depressive disorder: A meta-analysis of family high-risk studies. Compr. Psychiatry.

[41] Ranning A (2020). Morbidity and mortality in the children and young adult offspring of parents with schizophrenia or affective disorders-a nationwide register-based cohort study in 2 million individuals. Schizophr. Bull..

[42] Pierce M (2020). Effects of parental mental illness on children's physical health: Systematic review and meta-analysis. Br. J. Psychiatry.

[43] Petersen L, Mortensen PB, Pedersen CB (2011). Paternal age at birth of first child and risk of schizophrenia. Am. J. Psychiatry.

[44] Banks I, Baker P (2013). Men and primary care: Improving access and outcomes: MEN'S HEALTH. Trends Urol. Men's Health.

[45] Juel K, Christensen K (2008). Are men seeking medical advice too late? Contacts to general practitioners and hospital admissions in Denmark 2005. J. Public Health (Oxf.).

