# Supplementary material for: Social, demographic and health characteristics of men fathering children at different ages
Source: Sci Rep. 2021 Oct 25;11:21021. doi: 10.1038/s41598-021-00482-5 (PMC8545932; doi:10.1038/s41598-021-00482-5)
Supplement: Supplementary file 1 — Supplementary Information. [file 41598_2021_482_MOESM1_ESM.docx]

**Title:**

Social, demographic and health characteristics of men fathering children at different ages

All figures should be in colour.

**Authors:** Nina Kornerup*^a,b^, Anne-Marie Nybo Andersen^a^, Per Kragh Andersen^c^, Josephine Funck Bilsteen^a^ & SK Urhoj^a^

**Affiliations:**

a

University of Copenhagen

Faculty of Health and Medical Sciences

Section of Epidemiology

Øster Farimagsgade 5A, Box 2099

DK- 1014 Copenhagen K

b.

Department of Gynecology and Obstetrics

Holbæk Hospital
Smedelundsgade 60
DK-4300 Holbæk

c.

University of Copenhagen

Faculty of Health and Medical Sciences

Section of Biostatistics

Øster Farimagsgade 5 opg. B, P.O. Box 2099

DK-1014 Copenhagen K

**Authors' full name and contact information**

* Corresponding author:

Nina Kornerup, MD

E-mail: Ninakornerup@gmail.com

Phone: +45 4217 1936

Anne-Marie Nybo Andersen, MD, PhD

E-mail: [amny@sund.ku.dk](mailto:amny@sund.ku.dk)

ORCID: 0000-0002-4296-8488

Per Kragh Andersen, DrMedSci

E-mail: pka@biostat.ku.dk

Josephine Funck Bilsteen, PhD

E-mail: jfbi@sund.ku.dk

Stine Kjaer Urhoj, PhD

E-mail: stur@sund.ku.dk

| Supplementary Table I. Somatic and psychiatric diagnoses with ICD-10 and equivalent ICD-8 codes | | |
| --- | --- | --- |
| **Diagnosis** | **ICD-10** | **ICD-8** |
| Malignant and benign tumours | **All DC and DD codes** | 140.09 – 239.99 |
| Cardiovascular diseases | **All DI codes** | 400.09 – 458.99 |
| Endocrine diseases | **All DE** | 240.09 – 279.09 |
| Congenital anomalies | **All DQ** | 740.99 - 759.99 |
| Psychoactive substance abuse | **All F10-F19** | 303.09 - 304.99 |
| Schizophrenia and related disorders | **All F20-F29** | 295.09. 295.19. 295.29. 295.39. 295.59. 295.69. 295.89. 295.99. 297.09. 297.19. 297.99. 298.39. 296.89. 301.83 |
| Mood disorders | **Depression**  F32.3. F32.30. F32.31. F32.8. F32.9. F32.9A. F33. F33.0. F33.00. F33.01. F33.1. F33.10. F33.11. F33.2. F33.21. F33.3. F33.30. F33.31. F33.4. F33.8. F33.9 | 296.09. 296.29. 296.99. 298.09. 300.49 |
|  | **Bipolar disorders**  F30. F30.0. F30.1. F30.2. F30.20. F30.21. F30.8. F30.9. F31. F31.0. F31.1. F31.2. F31.20. F31.21. F31.3. F31.30. F31.31. F31.4. F31.5. F31.50. F31.51. F31.6. F31.7. F31.8. F31.9 | 296.19. 296.39 |
| Personality disorders | **F60** | 301.09 - 301.99 |
| Mental retardation | **F70-F79** | 310-315 |
| Pervasive developmental disorders: infantile  and atypical autism. Asperger's  syndrome. Pervasive developmental  disorder. Not otherwise specified (PDDNOS) | F84.0. F84.1. F84.10. F84.11. F84.12. F84.5. F84.8. F84.9 | 299.00. 299.01. 299.02. 299.03 |
| Behavioural and emotional disorders  with onset in childhood and  adolescence | **F90-F98** | 308.00 - 308.09; 306.29. 306.49 - 306.59 |

| **Supplementary table II, IRRs of fathering a child and the corresponding confidence intervals for sociodemographic and health characteristics according to conditions that prevailed in 2010 for all men aged 15-65 years living in Denmark January 1, 2011 divided into age groups.** | | | | | | | | | | | | | | | | | | | | |
| --- | --- | --- | --- | --- | --- | --- | --- | --- | --- | --- | --- | --- | --- | --- | --- | --- | --- | --- | --- | --- |
| Male age at January 1, 2011 (years) | | | | | | | | | | | | | | | | | | | | |
|  | 15-19 | | 20-24 | | | 25-29 | | 30-34 | | | 35-39 | | 40-44 | | 45-49 | | 50-54 | | 55-65 | |
| **IRs (CI) of fathering a child according to the father’s age by January 1, 2011 divided into age groups.** | | | | | | | | | | | | | | | | | | | | |
| IR/1000 pyrs | 6.85 | (6.68,7.03) | | 44.0 | (43.6,44.4) | 107.5 | (106.7,108.2) | | 99.0 | (98.3,99.6) | 46.1 | (45.7,46.6) | 14.6 | (14.4,14.8) | 4.33 | (4.20,4.46) | 1.25 | (1.18,1.33) | 0.26 | (0.24,0.28) |
| **IRRs (CI) for fathering a child for selected sociodemographic, socioeconomic and health characteristic according to the father’s age by January 1, 2011 divided into age groups.** | | | | | | | | | | | | | | | | | | | | |
| **Place of birth** | | | | | | | | | | | | | | | | | | | | |
| Denmark | 1 | (Ref.) | 1 | | (Ref.) | 1 | (Ref.) | 1 | | (Ref.) | 1 | (Ref.) | 1 | (Ref.) | 1 | (Ref.) | 1 | (Ref.) | 1 | (Ref.) |
| Other than Denmark | 1.34 | (1.25,1.44) | 1,03 | | (1.00,1.06) | 0,81 | (0.79,0.82) | 0,92 | | (0.90,0.94) | 1,37 | (1.33,1.41) | 2,40 | (2.31,2.50) | 3,76 | (3.53,4.02) | 5,45 | (4.82,6.16) | 6.97 | (5.75,8.43) |
| **Region of residents** | | | | | | | | | | | | | | | | | | | | |
| Capital Region | 1 | (Ref.) | 1 | | (Ref.) | 1 | (Ref.) | 1 | | (Ref.) | 1 | (Ref.) | 1 | (Ref.) | 1 | (Ref.) | 1 | (Ref.) | 1 | (Ref.) |
| Other Zealand | 1.43 | (1.32,1.55) | 1.39 | | (1.34,1.44) | 1.10 | (1.07,1.12) | 0.81 | | (0.79,0.83) | 0.64 | (0.62,0.66) | 0.59 | (0.56,0.62) | 0.54 | (0.49,0.59) | 0.47 | (0.39,0.57) | 0.59 | (0.44,0.78) |
| Southern Denmark | 1.37 | (1.27,1.48) | 1.36 | | (1.32,1.40) | 1.19 | (1.17,1.22) | 0.92 | | (0.91,0.94) | 0.72 | (0.70,0.74) | 0.59 | (0.56,0.62) | 0.56 | (0.51,0.61) | 0.48 | (0.40,0.57) | 0.59 | (0.46,0.76) |
| Central Jutland | 1.18 | (1.10,1.28) | 1.17 | | (1.14,1.21) | 1.20 | (1.17,1.22) | 1.01 | | (0.99,1.03) | 0.79 | (0.77,0.81) | 0.68 | (0.65,0.71) | 0.61 | (0.56,0.66) | 0.58 | (0.49,0.68) | 0.57 | (0.44,0.73) |
| Northern Jutland | 1.39 | (1.27,1.52) | 1.30 | | (1.26,1.35) | 1.16 | (1.13,1.19) | 0.90 | | (0.88,0.93) | 0.70 | (0.67,0.72) | 0.56 | (0.53,0.60) | 0.53 | (0.47,0.59) | 0.43 | (0.34,0.55) | 0.59 | (0.43,0.82) |
| **Educational level** |  |  |  | |  |  |  |  | |  |  |  |  |  |  |  |  |  |  |  |
| Primary and lower secondary (ISCED 1-2) | 0,42 | (0.06,2.99) | 0.78 | | (0.73,0.84) | 0.68 | (0.66,0.69) | 0.56 | | (0.55,0.57) | 0.62 | (0.60,0.64) | 0.70 | (0.66,0.73) | 0.76 | (0.69,0.83) | 0.67 | (0.57,0.80) | 0.76 | (0.58,1.00) |
| Upper secondary (ISCED 3) | 0,57 | (0.07,4.45) | 0.85 | | (0.79,0.92) | 0.93 | (0.91,0.95) | 0.78 | | (0.76,0.79) | 0.75 | (0.73,0.77) | 0.72 | (0.69,0.75) | 0.69 | (0.63,0.74) | 0.56 | (0.48,0.65) | 0.61 | (0.48,0.78) |
| Bachelor's degree (ISCED 5-6) | 1 | (Ref.) | 1 | | (Ref.) | 1 | (Ref.) | 1 | | (Ref.) | 1 | (Ref.) | 1 | (Ref.) | 1 | (Ref.) | 1 | (Ref.) | 1 | (Ref.) |
| Master's degree/doctoral level (ISCED 7-8) | - | - | 1.14 | | (0.81,1.61) | 1.16 | (1.12,1.20) | 1.17 | | (1.14,1.19) | 1.31 | (1.28,1.35) | 1.34 | (1.27,1.41) | 1.47 | (1.33,1.63) | 1.38 | (1.14,1.67) | 1.97 | (1.49,2.61) |
| **Employment** |  |  |  | |  |  |  |  | |  |  |  |  |  |  |  |  |  |  |  |
| Employed | 1 | (Ref.) | 1 | | (Ref.) | 1 | (Ref.) | 1 | | (Ref.) | 1 | (Ref.) | 1 | (Ref.) | 1 | (Ref.) | 1 | (Ref.) | 1 | (Ref.) |
| Self-employed | 0.81 | (0.53,1.25) | 1.08 | | (1.00,1.17) | 1.04 | (1.00,1.08) | 1.01 | | (0.98,1.04) | 1.10 | (1.06,1.14) | 1.21 | (1.14,1.28) | 1.46 | (1.33,1.61) | 1.62 | (1.35,1.96) | 1.81 | (1.39,2.34) |
| Student | 0.21 | (0.19,0.22) | 0.48 | | (0.46,0.49) | 0.72 | (0.71,0.74) | 0.95 | | (0.92,0.99) | 1.32 | (1.22,1.43) | 2.25 | (1.87,2.72) | 3.89 | (2.73,5.54) | 5.12 | (2.12,12.3) | 5.77 | (0.81,41.1) |
| Unemployed | 1.06 | (0.92,1.22) | 0.93 | | (0.89,0.97) | 0.58 | (0.56,0.60) | 0.60 | | (0.58,0.61) | 0.78 | (0.75,0.81) | 1.14 | (1.08,1.21) | 1.52 | (1.38,1.67) | 1.81 | (1.50,2.18) | 2.23 | (1.67,3.00) |
| Retired | 0.05 | (0.02,0.15) | 0.16 | | (0.13,0.20) | 0.15 | (0.13,0.17) | 0.20 | | (0.18,0.21) | 0.29 | (0.27,0.32) | 0.62 | (0.56,0.68) | 0.90 | (0.79,1.02) | 1.17 | (0.95,1.45) | 0.45 | (0.34,0.59) |
| Unattached to the labour market | 0.91 | (0.84,0.98) | 0.53 | | (0.50,0.55) | 0.38 | (0.37,0.40) | 0.52 | | (0.50,0.55) | 0.82 | (0.77,0.87) | 1.27 | (1.15,1.39) | 2.00 | (1.73,2.32) | 2.46 | (1.86,3.26) | 2.70 | (1.88,3.88) |
| **Disposable income** |  |  |  | |  |  |  |  | |  |  |  |  |  |  |  |  |  |  |  |
| Lower 10th percentile | 0.57 | (0.50,0.65) | 0.50 | | (0.47,0.53) | 0.52 | (0.51,0.54) | 0.76 | | (0.74,0.78) | 1.01 | (0.98,1.05) | 1.37 | (1.30,1.45) | 1.81 | (1.66,1.98) | 2.20 | (1.86,2.61) | 2.44 | (1.89,3.14) |
| 10-25th percentile | 0.56 | (0.50,0.63) | 0.70 | | (0.67,0.72) | 0.74 | (0.73,0.76) | 0.77 | | (0.75,0.79) | 0.88 | (0.85,0.90) | 1.11 | (1.06,1.16) | 1.35 | (1.24,1.47) | 1.67 | (1.42,1.96) | 1.12 | (0.84,1.49) |
| 25-75th percentile | 1 | (Ref.) | 1 | | (Ref.) | 1 | (Ref.) | 1 | | (Ref.) | 1 | (Ref.) | 1 | (Ref.) | 1 | (Ref.) | 1 | (Ref.) | 1 | (Ref.) |
| 75-90th percentile | 2.34 | (2.19,2.50) | 1.64 | | (1.59,1.68) | 1.29 | (1.27,1.31) | 1.21 | | (1.19,1.24) | 1.14 | (1.11,1.17) | 1.08 | (1.03,1.13) | 1.05 | (0.95,1.15) | 0.93 | (0.76,1.13) | 1.40 | (1.08,1.83) |
| Upper 90th percentile | 3.92 | (3.68,4.17) | 2.17 | | (2.11,2.24) | 1.38 | (1.35,1.41) | 1.29 | | (1.27,1.32) | 1.26 | (1.22,1.30) | 1.21 | (1.15,1.28) | 1.29 | (1.17,1.43) | 1.59 | (1.32,1.92) | 2.01 | (1.54,2.62) |
| **Somatic diseases** | | | | | | | | | | | | | | | | | | | | |
| Malignant and benign tumours | 0.95 | (0.83,1.09) | 0.98 | | (0.93,1.03) | 1.04 | (1.00,1.07) | 0.98 | | (0.96,1.02) | 0.94 | (0.90,0.98) | 0.91 | (0.86,0.97) | 0.78 | (0.70,0.88) | 0.91 | (0.75,1.10) | 0.72 | (0.55,0.95) |
| Cardiovascular diseases | 1.23 | (1.05,1.45) | 1.11 | | (1.05,1.17) | 1 | (0.97,1.04) | 0.93 | | (0.90,0.95) | 0.90 | (0.87,0.93) | 0.86 | (0.81,0.91) | 0.91 | (0.84,0.99) | 0.82 | (0.70,0.95) | 0.64 | (0.52,0.79) |
| Endocrine diseases | 1.04 | (0.93,1.17) | 0.88 | | (0.83,0.92) | 0.84 | (0.81,0.87) | 0.81 | | (0.78,0.84) | 0.76 | (0.72,0.80) | 0.82 | (0.76,0.89) | 1.08 | (0.96,1.20) | 1.01 | (0.83,1.23) | 0.73 | (0.55,0.96) |
| Congenital anomalies | 0.91 | (0.84,0.99) | 0.91 | | (0.88,0.95) | 0.93 | (0.91,0.95) | 0.93 | | (0.91,0.95) | 0.91 | (0.88,0.94) | 0.88 | (0.81,0.95) | 1.12 | (0.97,1.30) | 0.87 | (0.61,1.25) | 0.82 | (0.45,1.49) |
| **Psychiatric illnesses** | | | | | | | | | | | | | | | | | | | | |
| Psychoactive substance abuse | 2.77 | (2.48,3.09) | 1.20 | | (1.15,1.25) | 0.79 | (0.77,0.82) | 0.68 | | (0.66,0.70) | 0.71 | (0.67,0.74) | 0.69 | (0.64,0.75) | 0.63 | (0.54,0.73) | 0.54 | (0.40,0.73) | 0.55 | (0.34,0.89) |
| Schizophrenia and related disorder | 1.66 | (1.27,2.15) | 0.60 | | (0.54,0.68) | 0.36 | (0.33,0.39) | 0.34 | | (0.31,0.37) | 0.40 | (0.36,0.45) | 0.54 | (0.45,0.64) | 0.95 | (0.75,1.20) | 0.53 | (0.27,1.02) | 0.75 | (0.28,2.00) |
| Depression | 1.27 | (1.01,1.59) | 0.92 | | (0.85,1.00) | 0.62 | (0.58,0.66) | 0.59 | | (0.55,0.62) | 0.70 | (0.65,0.75) | 0.87 | (0.78,0.97) | 1.30 | (1.11,1.53) | 1.35 | (1.00,1.84) | 1.62 | (1.05,2.51) |
| Bipolar disorders | 0.42 | (0.06,2.99) | 0.69 | | (0.49,0.97) | 0.51 | (0.42,0.63) | 0.44 | | (0.36,0.53) | 0.76 | (0.63,0.93) | 0.59 | (0.41,0.85) | 0.36 | (0.16,0.80) | 0.80 | (0.30,2.13) | 0.41 | (0.06,2.95) |
| Personality disorders | 2.31 | (1.62,3.29) | 0.99 | | (0.88,1.10) | 0.55 | (0.51,0.59) | 0.50 | | (0.47,0.54) | 0.59 | (0.54,0.65) | 0.67 | (0.57,0.79) | 1.08 | (0.85,1.38) | 1.40 | (0.88,2.23) | 1.36 | (0.56,3.27) |
| Mental retardation | 0.84 | (0.66,1.08) | 0.41 | | (0.35,0.48) | 0.21 | (0.18,0.25) | 0.14 | | (0.11,0.18) | 0.08 | (0.05,0.13) | 0.05 | (0.02,0.17) | 0.23 | (0.09,0.62) | 0.27 | (0.04,1.89) | - | - |
| Pervasive developmental disorders | 0.40 | (0.31,0.52) | 0.32 | | (0.27,0.37) | 0.16 | (0.12,0.20) | 0.15 | | (0.10,0.21) | 0.08 | (0.04,0.20) | - | - | - | - | - | - | - | - |
| Behavioural and emotional disorders | 1.73 | (1.60,1.88) | 0.90 | | (0.85,0.94) | 0.57 | (0.54,0.60) | 0.47 | | (0.44,0.50) | 0.61 | (0.56,0.66) | 0.75 | (0.65,0.86) | 1.13 | (0.91,1.41) | 0.82 | (0.47,1.41) | 0.78 | (0.29,2.08) |
